# Influence of a Cell-Phone Conversation on Balance Performance in Women with Fibromyalgia: A Cross-Sectional Descriptive Study

**DOI:** 10.1155/2019/5132802

**Published:** 2019-11-11

**Authors:** Santos Villafaina, Narcis Gusi, Sandra Rodriguez-Generelo, Juan de Dios Martin-Gallego, Juan Pedro Fuentes-García, Daniel Collado-Mateo

**Affiliations:** ^1^Physical Activity and Quality of Life Research Group (AFYCAV), Faculty of Sport Science, University of Extremadura, Cáceres, Spain; ^2^EXERNET Research Network on Exercise and Health in Special Populations, Spain; ^3^Faculty of Sport Science, University of Extremadura, Cáceres, Spain; ^4^Centre for Sport Studies, Rey Juan Carlos University, Fuenlabrada, Madrid, Spain

## Abstract

**Background:**

Altered balance is a common and debilitating symptom of fibromyalgia. Previous studies have investigated balance under single-task conditions that do not reflect real-life situations. The present study evaluated the impact of a simultaneous cell-phone conversation on balance performance in a case-control cohort from Spain.

**Methods:**

A cross-sectional case-control study was performed in 34 women recruited from local self-help organizations and university facilities (*n* = 18 fibromyalgia cases; *n* = 16 healthy, pain-free controls). Participants performed the 30 s postural stability test, as implemented in the Biodex Balance System, under two conditions: (a) as a single task and (b) as a dual task, i.e., while holding a simultaneous cell-phone conversation with a technician. Intergroup differences in dual task costs were calculated.

**Findings:**

Compared with controls, women with fibromyalgia showed higher dual-task costs in balance variables, such as overall sway (*pp* value = 0.039) and anterior/posterior sway (*p* value = 0.007). In the dual-task condition, overall (*p* value = 0.004) and anterior/posterior (*p* value = 0.012) sway indices significantly decreased in women with fibromyalgia but not in controls.

**Interpretation:**

In women with fibromyalgia, balance performance was adversely impacted by the holding of a simultaneous cell-phone conversation. The inability to conduct two tasks simultaneously may be related to reduced attention and may increase the risk of falling in this population.

## 1. Introduction

Women with fibromyalgia report poor health-related quality of life and a decreased ability to perform the activities of daily living [1, 2]. This may result from the multitude of potential fibromyalgia symptoms, which include pain, stiffness, fatigue, unrefreshing sleep, poor cardiovascular fitness, cognitive impairments, memory and attentional deficits, depression/anxiety, and poor balance [3–5].

Altered balance is one of the ten most common and debilitating symptoms of fibromyalgia [6]. Adequate balance is crucial for the safe performance of daily motor activities, since it reduces the risk of falling. Previous studies showed that women with fibromyalgia have an impaired balance and an increased risk of falling, these two characteristics being correlated [4, 7, 8]. In this regard, a previous study showed that rate of torque development of the hip extensors, duration of symptoms, and pain were the main predictors of falling in women with fibromyalgia [9].

However, most methods used to evaluate physical function in research—in particular balance—do not reflect real-life situations, since they are performed under conditions in which the participant's full attention is focused on a single specified task. In this single-task approach, the participant is required to perform a balance task as concentrated as possible. To render the evaluation of physical function more ecological and representative of real-life conditions, previous authors have proposed the simultaneous performance of two tasks, such as walking while holding a conversation [10, 11].

Although women with fibromyalgia may not display hypervigilance for a somatosensory stimuli under dual-task condition [12], previous studies have evaluated the impact of a simultaneous cognitive or affective task on motor task performance in women with fibromyalgia [13–16]. Among other, de Gier et al. [16] showed that participants tolerated physical activity in the dual task longer than in the single task. Furthermore, Villafaina et al. [15] showed a decrease in the arm curl test performance as well as a reduction in the range of movement in the dual-task condition that involved the remembering of three random unrelated words. Villafaina et al. [14] also reported a reduction in stair climbing performance using that same cognitive task. Similarly, Sempere-Rubio et al. [13] found that balance performance was affected by recalling the events of a stressful day.

In modern society, one of the most representative and ecological secondary tasks is the holding of a cell-phone conversation while performing a motor task. The adverse impact of a cell-phone conversation on driving performance is well documented [17]. This research has shown that cell-phone conversations during driving are associated with a decrease in reaction times and stimulus detection, despite the employment of hand-free devices and compensatory strategies such as speed reduction. A cell-phone conversation involves motor and cognitive demands that negatively affect cognitive processing performance [18] and which increase the rate of errors during the performance of continuous mental arithmetic [19]. In tasks involving a motor component, such as crossing the street, a conversation affects both gait and attention, thus increasing the risk of accidents [20].

The aim of the present cross-sectional study was to evaluate the impact of a simultaneous cell-phone conversation on balance performance in women with fibromyalgia and healthy pain-free controls.

## 2. Materials and Methods

### 2.1. Participants

The cohort comprised 34 women from Spain: *n* = 18 fibromyalgia cases; *n* = 16 healthy, pain-free controls. The Extremadura Association of Fibromyalgia (AFIBROEX) in Cáceres (Spain) recruited women with fibromyalgia by telephone calls in March 2018. Inclusion criteria for fibromyalgia cases were as follows: (a) rheumatologist-assigned diagnosis of fibromyalgia in accordance with the 2010 criteria of the American College of Rheumatology (2) and (b) ability to stand without support.

Women without fibromyalgia, who were matched in terms of age, also participated in the present study. They were recruited in the University facilities or were close to patients. Participants were contacted and given an appointment based on the time availability.

Exclusion criteria for patients and controls were as follows: (a) age >70 years; (b) pregnancy; (c) presence of a severe visual or hearing impairment; and (d) vestibular disturbance.

The study protocol was approved by the Committee of Bioethics of the University of Extremadura. All subjects provided written informed consent prior to inclusion.

### 2.2. Procedure

The study was conducted between March and June 2018 at the Faculty of Sport Science in the University of Extremadura. For each participant, height was measured, and data were collected concerning age and physical health. Women with fibromyalgia also completed the Spanish version of the revised the Fibromyalgia Impact Questionnaire (FIQ-R) [21, 22] and a Visual Analogue Scale (VAS) for pain intensity.

Each participant was asked to perform a balance task under two conditions: (a) single task, i.e., with no simultaneous task, and (b) dual task, i.e., while holding a cell-phone conversation with a technician (who was blinded for group allocation). To avoid interference between tasks as a consequence of learning and familiarization, the order of these two tasks was randomized.

The dual-task consisted in an active cell-phone conversation. In order to ensure that participants maintained an active conversation, the cell-phone call always started by “*Hello! What did you do the last weekend?*.” If the participants did not take all the time with the previous question, the technician asked more questions such as: *“What is the dedication of your son/daughter/couple?” “Where do you live?” “What did you do yesterday?”* and *“Have you ever lived in other place?”*

Balance tasks were conducted using the Biodex Balance System (Shirley, NY, USA), which is widely used in the evaluation and training of balance [23]. For the purposes of the present study, the postural stability test was selected, since this emphasizes the ability to maintain a center of balance, and the degree of stability can be altered during the course of the task. The duration of the test was 30 s. The platform was stable at the beginning of the task and is progressively becoming more unstable until the end of it, thus ensuring that the participant was required to adjust their posture. Participants were able to see their center of gravity in a screen grid, so they had visual feedback. A 45 s rest period was inserted between the two task conditions (single and dual). In accordance with the findings and suggestions of a previous study, the position of the feet was controlled using adhesive footprints [24]. The sway index, which is the root mean square distance for the *X*, *Y* coordinates over the course of the 30 s test [25], was used in the statistical analyses because it does not depend on the starting distance from the center of the platform.

### 2.3. Statistical Analysis

All analyses were conducted using the Statistical Package for Social Science (version 20.0, SPSS , Inc., Chicago, IL). Results from Shapiro–Wilk and Kolmogorov–Smirnov tests were used to determine the requirement for parametric or nonparametric statistics. The balance performance data did not follow a normal distribution, and nonparametric tests were therefore selected. The Mann–Whitney *U* test and the Wilcoxon signed-rank test were used to determine intergroup and intragroup differences, respectively. The Mann–Whitney *U* test was used to compare women with fibromyalgia and healthy controls in the single- and dual-task conditions (fibromyalgia *vs* control). The Wilcoxon signed-rank test was used to evaluate the effect of adding the cell-phone task in each group (single *vs* dual).

Dual-task cost (DTC) was computed as follows: DTC = (dual task − single task)/single task. The interaction effects of task (single *vs* dual) and group (fibromyalgia *vs* control) were evaluated via intergroup comparisons using the Mann–Whitney *U* test. Nonparametric effect size *r* was calculated [26]. Values of 0.37, 0.24, and 0.10 represent large, medium, and small effect sizes, respectively [27].

The alpha-level of significance was set at 0.05.

## 3. Results

The main characteristics of the participants are summarized in Table 1. Mean (SD) age was 54.83 (8.98) in the fibromyalgia group and 58.43 (10.55) in controls. No significant intergroup differences were found for age or height. In women with fibromyalgia, the mean (SD) FIQ-R score was 58.71 (20.85).

### 3.1. Intragroup Analyses

The Wilcoxon signed-rank test was used to explore differences between single and dual tasks for each group (fibromyalgia and control groups). For the fibromyalgia group, intragroup analyses revealed a significant increase in the sway index in the dual-task condition. This affected the variables “overall sway index” and “forward/backward sway index” (anterior/posterior). The variable “left/right sway index” (medial/lateral) was not significantly influenced by the dual-task condition. In controls, no significant differences were observed between the single-task and dual-task conditions (Table 2).

### 3.2. Intergroup Analyses

The Mann–Whitney *U* test did not show differences between groups for either the single- or the dual-task conditions. However, when DTC was calculated ((dual task − single task)/single task), significant differences were found. In this regard, the overall sway index and the anterior/posterior sway index were higher in women with fibromyalgia than in controls (Table 3).

## 4. Discussion

The main finding of the present study was that a cell-phone conversation had a significant impact on the DTC of the overall sway index and the anterior/posterior sway index of women with fibromyalgia when compared with age-matched controls. In this line, the conversation had a negative impact on the anterior/posterior and overall balance in women with fibromyalgia but not in healthy controls. To our knowledge, this is the first study to evaluate balance in this population during the performance of a typical activity of daily living.

Our results showed that differences in balance between women with fibromyalgia and controls are only significant when analysing the degree of performance decline that is prompted by the secondary task. This is relevant since activities of daily living usually are presented as two or more task simultaneously and, therefore, women with fibromyalgia could have problem to develop these activities. In this regard, our results showed that the DTC for overall balance was higher in women with fibromyalgia than in age-matched controls. This could be the reason why women with fibromyalgia are more prone to falling than healthy, age-matched women since their balance, strength, and functionality levels are similar to those of older women [4, 28], and this reduced functional performance, along with the other symptoms of fibromyalgia, may predict the number of falls [9]. Additionally, we also found significant differences in the anterior/posterior DTC sway index, which has been related to fall risk in older adults [29, 30].

We attempted to induce changes in balance by implementing a dual-task paradigm which includes a motor task (balance task with instability) and a cognitive demanding task (an active cell-phone conversation). In this regard, previous studies in women with fibromyalgia have shown that physical performance is reduced under dual-task paradigm compared with age-matched controls. Concerning balance, Sempere-Rubio et al. [13] investigated the performance under dual-task condition and found that balance was affected by recalling the events of a stressful day. Our results are in line with that research, but this is the first study reporting that a typical activity of daily living (a cell-phone conversation) can reduce the overall balance in women with fibromyalgia.

Although the interference of the cognitive task on the motor task has been studied, in our study, the cell-phone performance was not collected, so the interpretation of prioritization and bidirectional interference is limited. The previous studies showed how older adults prioritized the task that they perceived to have greater importance [31, 32]. Thus, they usually prioritize the postural control due to the high prevalence of instability and risk of falling [31, 33]. Therefore, taking into account the balance task (where the task commenced with a stable surface and ends with an unstable surface) and the poor balance of women with fibromyalgia [4], hypothetically participants would prioritize the balance task over the cognitive task. This is also relevant because, although prioritizing the motor task, the balance performance decreased more than in healthy controls. In this regard, a previous study showed that patients with fibromyalgia presented an altered attention [34], which may be related to the close link between attention and pain [35]. Thus, it is possible that women with fibromyalgia are constantly focused on pain, and therefore the attention available for other tasks is limited (explaining the poor performance during dual-task conditions). This hypothesis is supported by the findings of a recent study in chronic pain patients, which showed that a reduction in pain may reduce the DTC [36]. However, further studies are needed to confirm these hypotheses.

The present study used a common task that involves both motor [37] and cognitive demands [38]. This secondary task was selected as it goes beyond artificial “verbal tasks” that may fail to mimic the features of a real daily activity [19]. Therefore, some clinical implications emerged from our results. First, given the described interference of cognitive tasks on motor simultaneous tasks, the addition of secondary tasks in regular physical training may lead to further benefits in the daily living of women with fibromyalgia. Moreover, balance tasks should be focused in the frontal plane, as our results suggest that it is the most affected plane by an active conversation and previous research has identified a relation between that variable and falling risk [29, 30]. However, the addition of simultaneous tasks should not be limited to the physical training, but also it could be used as evaluation measure, providing valuable information about the performance of a person in their daily life. Furthermore, previous studies have shown that dual-task evaluation is more useful than single-task evaluation in terms of assessing future fall risk in older adults [39, 40].

The present study had three main limitations. First, the sample size was relatively small, which may explain the lack of significant intergroup differences in single- and dual-task balance performance. Second, to increase the homogeneity of the sample, the cohort was restricted to women aged ≤70 years. Therefore, the results cannot be generalized to women aged >70 years or to males. Third, conversation performance was not registered. This issue impaired the study of the task prioritization of women with fibromyalgia.

## 5. Conclusion

Women with fibromyalgia experienced a more pronounced reduction in balance performance than healthy controls when performing a simultaneous secondary task. Specifically, an increase in both overall and anterior/posterior sway was observed when a secondary task (an active cell-phone conversation) was added. This secondary simultaneous task represents a real activity of daily life that is often performed while performing another motor task, such as walking or standing. The present data will facilitate understanding of the risk of falling in women with fibromyalgia.

## Figures and Tables

**Table 1:** Demographic characteristics of the sample and intergroup comparisons.

	Women with fibromyalgia (*n* = 18)	Healthy controls (*n* = 16)	*p* value
Age	54.83 (8.99)	58.44 (10.55)	0.237
Stature	159.78 (6.62)	160.06 (4.73)	0.772
Pain intensity (VAS 0-100)	62.78 (17.76)	N/A	—
Duration of symptoms (years)	17.75 (13.26)	N/A	—
FIQ-R	58.71 (20.85)	N/A	—
Single-task condition			
Overall sway index	1.37 (0.93)	1.37 (0.93)	0.878
Anterior/posterior sway index	1.06 (0.46)	1.66 (2.05)	0.347
Medial/lateral sway index	1.43 (1.11)	1.22 (1.17)	0.237
Dual-task condition			
Overall sway index	1.97 (1.17)	1.52 (1.21)	0.075
Anterior/posterior sway index	1.64 (0.62)	1.24 (0.66)	0.064
Medial/lateral sway index	1.62 (1.22)	1.42 (1.37)	0.198

Results are shown as mean (standard deviation). FIQ-R: revised version of the Fibromyalgia Impact Questionnaire; N/A: not applicable. *p* values refer to the Mann–Whitney *U* test.

**Table 2:** Intragroup single- *vs* dual-task comparisons for women with fibromyalgia and healthy controls.

Variable	Intragroup *p* value	Effect size (*r*)
Women with fibromyalgia		
Overall sway index	0.004	0.67
Anterior/posterior sway index	0.012	0.59
Medial/lateral sway index	0.286	0.25

Healthy controls		
Overall sway index	0.349	0.23
Anterior/posterior sway index	0.438	0.19
Medial/lateral sway index	0.155	0.36

*p* values refer to the Wilcoxon signed-rank test.

**Table 3:** Comparison of dual-task costs between women with fibromyalgia and healthy controls.

	Women with fibromyalgia (*n* = 18)	Healthy controls (*n* = 16)	*p* value	Effect size (*r*)
DTC overall sway index	0.64 (1.00)	0.10 (0.47)	0.039	0.36
DTC anterior/posterior sway index	0.89 (1.15)	0.03 (0.60)	0.007	0.46
DTC medial/lateral sway index	0.52 (1.13)	0.35 (0.80)	0.798	0.04

DTC: dual-task cost = (dual task − single task)/single task. *p* values refer to the Mann–Whitney *U* test.

## Data Availability

Data used to support the study will be available upon request.
